# Supplementary material for: Slow waves in locally resonant metamaterials line defect waveguides
Source: Sci Rep. 2017 Nov 8;7:15105. doi: 10.1038/s41598-017-15403-8 (PMC5678171; doi:10.1038/s41598-017-15403-8)
Supplement: Supplementary file 1 — Supplementary information [file 41598_2017_15403_MOESM1_ESM.pdf]

Supplementary information

Slow waves in locally resonant metamaterials line defect waveguides

Nadège Kaina[1], **Alexandre Causier**[2], **Yoan Bourlier**[2], **Mathias Fink**[1], **Thomas Berthelot**[2], **Geoffroy Lerosey**[1]

[1] *Institut Langevin, ESPCI ParisTech & CNRS, Paris, France*

[2] *CEA Saclay, IRAMIS, NIMBE, LICSEN, UMR 3685, F-91191, Gif sur Yvette, France*

1 Sample fabrication

Thickness and surface morphology of the plated copper layers were examined with a SEM, scanning electron microscope, (JSM-5510LV, JEOL, Tokyo, Japan). Wires of the metamaterial were cryo-fractured with liquid nitrogen to probe the height of the copper layer. SEM micrographs of the wire slice were taken at various magnifications ranging from 100× to 5 000× (X300 displayed in Fig. S1) applying the secondary electron detector. The acceleration voltage and working distance were respectively 4 kV and 17 mm. From Figure S1, we clearly observe that the copper layer is between 20 µm and 50 µm high, largely over the copper skin depth at 5 GHz (0.92 µm).

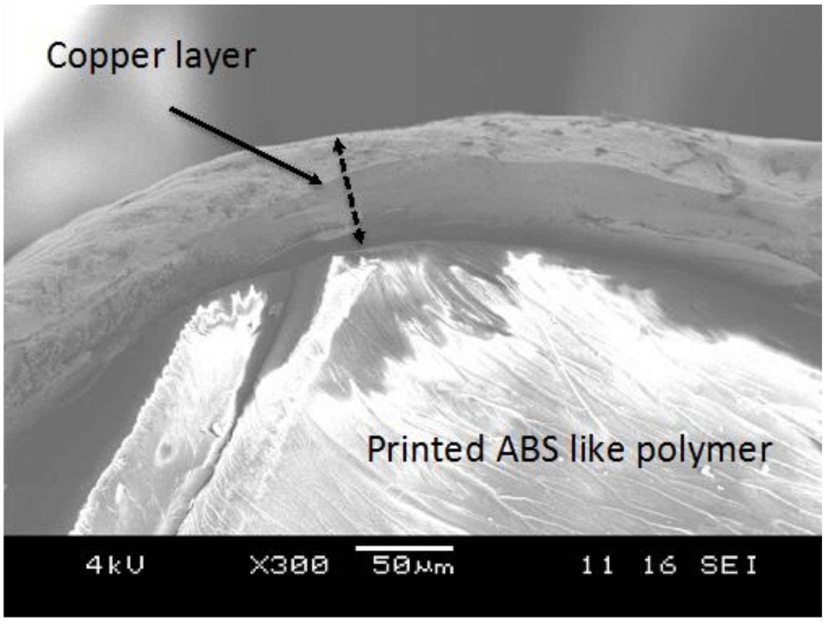

Figure S1| SEM image of a part of a section of a polymer copper coated wire after the whole metallization process.

2 Design of the devices

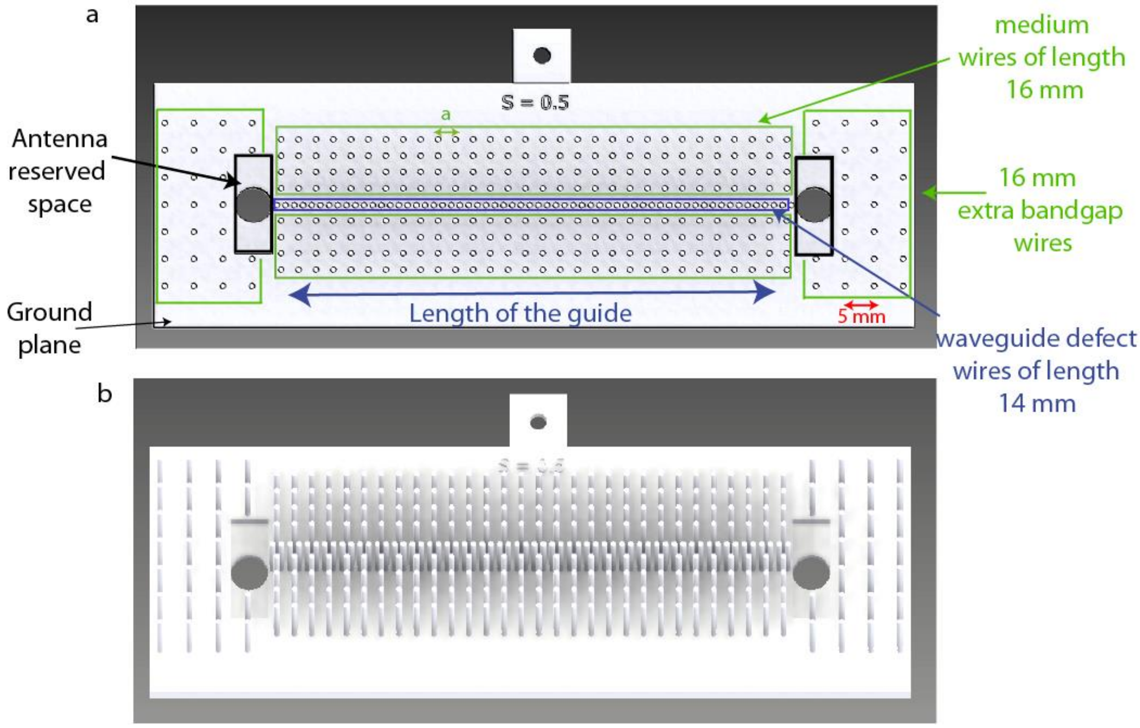

Figure S2| CAO views of a typical device. **a**, top, **b**, perspective

The devices all consist of a ground plane (height = 3 mm, length and width variable) on which two 6.5x18 mm^2 rectangles are extruded, corresponding to the required space to put the antenna base. Within this space, a hole is drilled to insert the SMA connector so that the electrical wires for the measurements are connected below the ground plane.

The wires of the medium (green in Fig. S2) are all of length L = 16 mm and surround the waveguide in the whole space between the antennas. The period *a* of this medium depends on the device and varies from 2 mm to 10 mm. The waveguide is composed of N (60 for the devices of Figure 3, 26 for the ones of Figure 4) wires of length L_d = 14 mm, separated by a distance a_g (from 1.5 mm to 6.97 mm). All the wires are 1 mm wide, which is limited by the resolution of the 3D printer to grow wires with conformal width on such heights.

Some extra wires of length L (green in Fig. S2) arranged on a 5 mm lattice are added behind the antennas in order to provide a bandgap medium at the waveguide transmission band frequencies, so that the antennas are isolated from the exterior. It then insures noise limited measurements.

3 Simulation of the transmission through the tortuous waveguide

Using the software CST Microwave studio, we simulate the transmission through a sample analogous to the experimental tortuous waveguide of the figure 1 in the main text. The S-parameter through the waveguide is displayed in figure S3 (red). We observe the same form of transmission spectrum as in the experiment, with low frequency modes and a bandgap attributed to the environing wire medium and a transmission band around 5 GHz. To demonstrate that this transmission band is indeed the signature of the transmission through the line defect waveguide, we furthermore simulate the same devices but with the wires that all have the same length (as schemed in figure S3), that is that we suppress the line defect waveguide. The transmission, in black, confirms that the transmission band disappears and that only the bandgap of the medium remains for this frequency range.

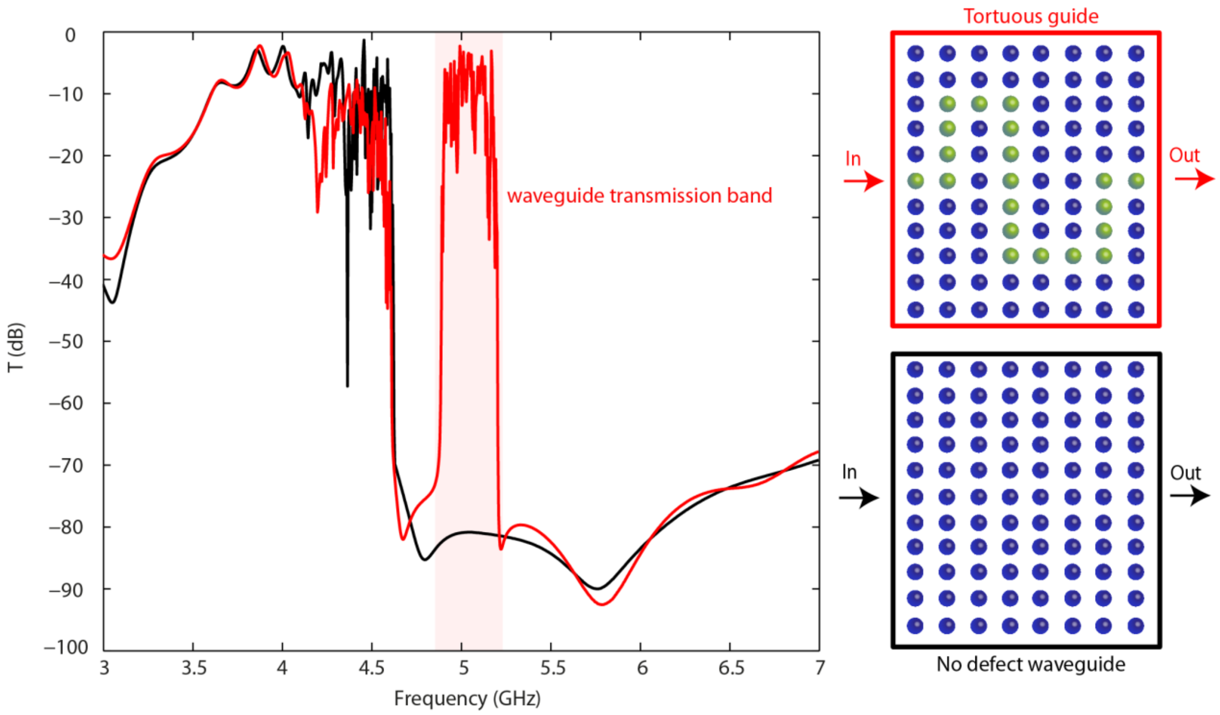

Figure S3|Simulated transmission through the tortuous waveguide of Figure 1. Simulated transmission through the waveguide (red) and through the medium with no defect line waveguide (black) along with schematics of the two simulated devices (blue dots = wires of the medium, green dots = defect wires).

4 Homemade antennas

Homemade antennas were used for all measurements (Fig. S4). In both spectral and temporal measurements, the antennas consisted of cut stain wires soldered on SMA connectors. The length of the antenna wires was manually adapted in order to get the best possible coupling to the waveguide, that is the highest transmission (considering the losses) and the minimum oscillations (typical of reflections). The length was set to 21 mm. The antennas were placed at the designed spot within the

device at each side of the waveguide, around 4 mm away from the first wire of the waveguide, distance that is limited by the width of the SMA base. Since this distance partly drives the coupling to the waveguide (the further, the lower the coupling), we soldered a stain wire of length 14 mm (same as for the defects) on the base of the SMA so that it decreases the distance in-between the antenna and the first wire.

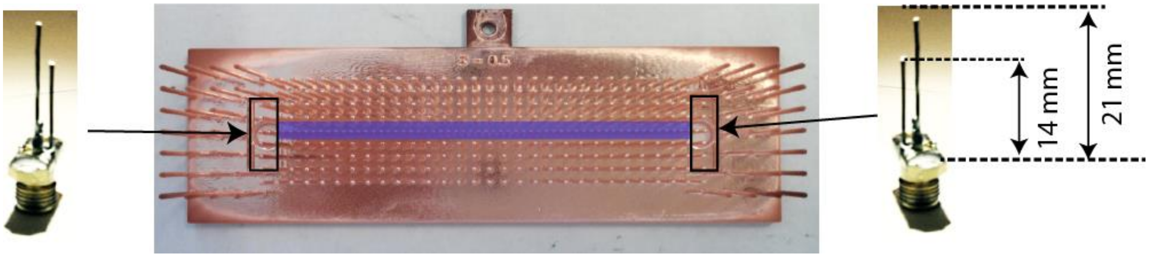

Figure S4| Picture of a device and of the homemade antennas. The waveguide defect wires are highlighted in blue and the holes for antennas in solid black rectangles. Photographs of the home made antennas used for spectral and temporal measurements with the feed antenna of 21 mm and the extra defect wire of 14 mm (sides).

5. Dispersion relation measurements set-up

Measurement set-up for the dispersion relation of the line defect metamaterial waveguides.

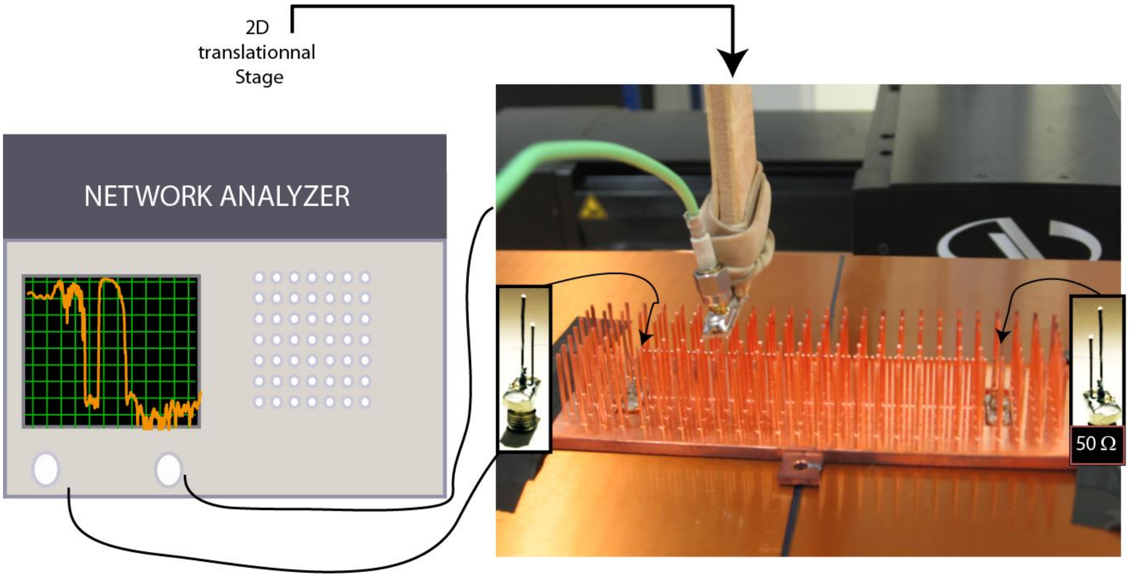

Figure S5| Setup for the measurements requiring scanning of the E-field over the device.

5 Supplementary experimental results

a. Wide band temporal measurement

We know from the shape of the dispersion relation, relatively similar to a tight-binding one that the group index consequently varies within the waveguide transmission band. For the sake of clarity, in the main text, we restrained our measurement to n_g around central frequencies (averaged over 20% of the bandwidth, see shaded areas in Fig. S6) though it would obviously give the lower delays.

In order to probe the properties over the whole bandwidth of the waveguide, we sent for each device a short broadband pulse centered on the latter central frequency. The measurements were then filtered on a 20 MHz bandwidth for 200 different frequencies within the waveguide transmission band (results are displayed on Fig. S6). The group index for each frequency was retrieved from the delay between the sent and received filtered pulses. It was measured from the maxima of the envelope of those pulses while the reference velocity was taken as $3e^8$ m/s. The amplitudes of the pulses were normalized to the maximum of amplitude of the input pulses.

The group index indeed dramatically increases at the edges of the band where the dispersion curve flattens. The group index while plotted on the whole frequency range shows that much higher values can be reached by changing the operating frequency (up to 300 for a = 2 mm dense medium) as displayed in Figure S6a. Of course, the group index enhancement will lead to an increase of the transmission losses, so that there is a trade-off to find to get the most efficient delay lines.

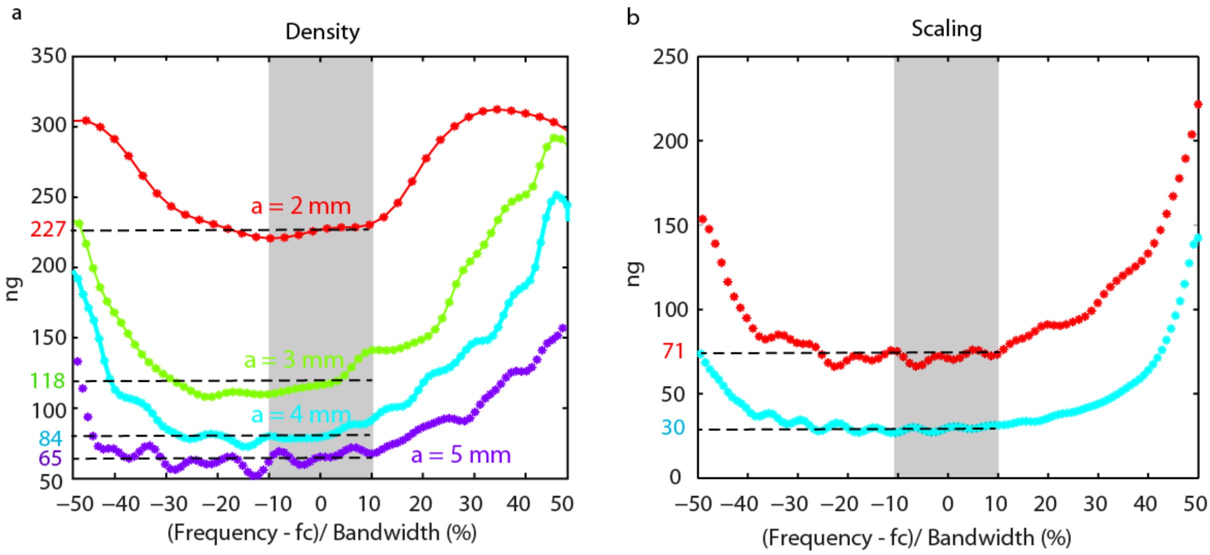

Figure S6| Experimental broadband measurements of the group index. a, samples of Figure 3a and **b**, samples of Figure 4b. Data are plotted as a function of the frequency, centered on f_c and normalized by the bandwidth. The x-axis is presented as a percentage of the bandwidth. The frequency range on which the group index displayed in the main text is averaged is shaded in grey, while the corresponding value is advised on the y-axis.

We furthermore emphasize that this curves are not entirely symmetric since our dispersion relations are not purely tight-binding but display some features of a polariton. Particularly, the group index is higher at the upper edge of the band, where the dispersion gets to its flat asymptote which is

characteristic of polaritonic dispersion relations. Finally, we observe that the larger the periodicity of the medium, the larger the bandwidth on which the group index is relatively constant, that is the bandwidth with linear dispersion.

b. Broadband pulse transmission

In the manuscript, the temporal pulse measurements displayed are narrow filtered on a 20 MHz bandwidth around the central frequency, to enable a quantitative estimation of the group index. However, by consequently broadening the pulses, this hides the fact that the delays achieved by the line defect waveguides are several pulse length long and hence that the received and sent pulses do not overlap. This is evidenced in figure S7, showing the transmission of short pulses, centered on the central frequency, and whose length correspond to the total bandwidth for each experimental waveguide of Figure 2 in the manuscript. The delays range from 6.5 to 11.6 of the sent Gaussian pulses FMHW. Naturally, because of the dispersion over the frequency bandwidth that was previously discussed, we see that the broadband output pulses are consequently distorted.

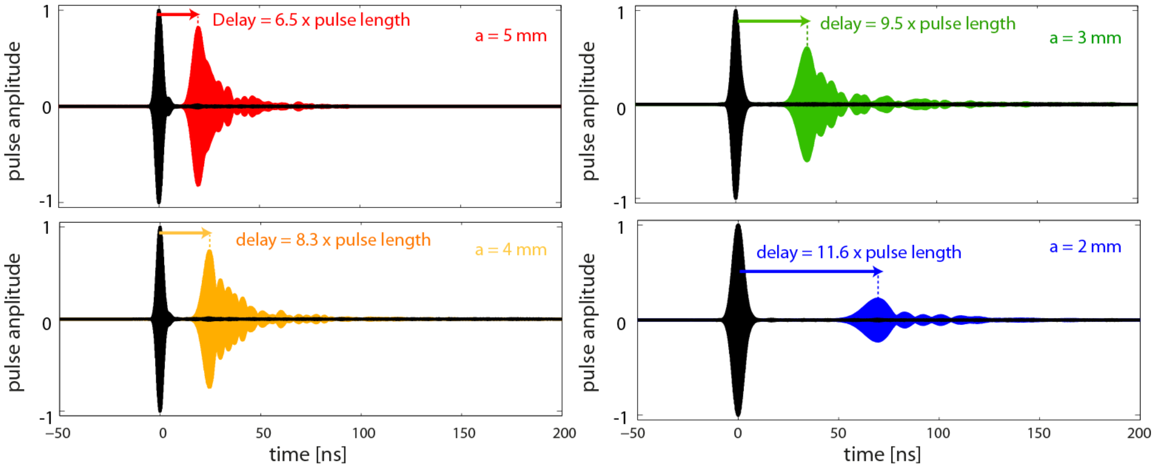

Figure S7| Experimental transmissions of short pulses for the waveguides of Figure 2. Short pulses measured in input (black) and output (color) of each waveguides, centered on the central frequency and Gaussian filtered on the total bandwidth of the corresponding waveguide.

c. Estimation of the losses

Attenuation is an important matter, especially when dealing with metallic structures and slow wave propagation. For the metamaterial line defect waveguides presented in the paper (Figure 2), the transmitted amplitude is comprised between -1.8 dB and -13 dB after a propagation through the Lg = 1.6 λ long waveguide. Those losses are due to the intrinsic properties of the plated copper and we see in figure S8 that (apart from the a = 2 mm sample that suffered from a lower quality plating), the

losses seem linear with the inverse of the material density, i.e. logically proportional to the time spent by the wave in the waveguide. Though the attenuation is not negligible, it has to be compared with other conventional microwave waveguides as coaxial cables or rigid rectangular waveguides. In figure S8, we see that the attenuation of our waveguides varies from 0.1 to 0.2 dB/ns, which is, around 5 GHz, lower than actual implementations. Note that microstrip lines, commonly implemented for microwave devices (including delay lines) suffer from losses even tenfold larger.

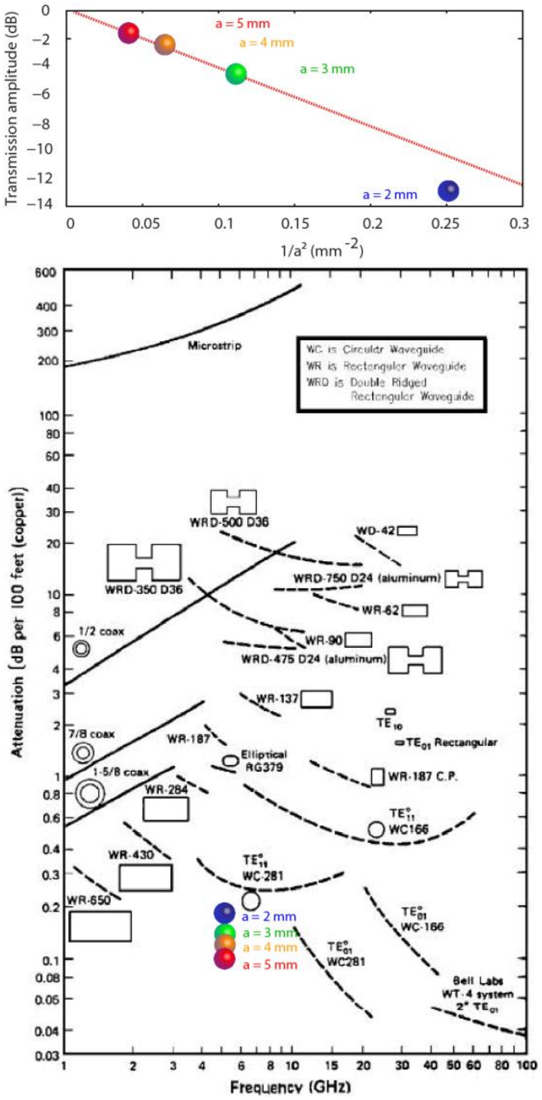

Figure S8│ Experimental transmissions amplitudes for the waveguides of Figure 2. Transmission amplitudes after propagation in the waveguides and comparison with common attenuation in microwave waveguides
